# Supplementary material for: Generic predictions of output probability based on complexities of inputs and outputs
Source: Sci Rep. 2020 Mar 10;10:4415. doi: 10.1038/s41598-020-61135-7 (PMC7064605; doi:10.1038/s41598-020-61135-7)
Supplement: Supplementary file 1 — Supplementary Information. [file 41598_2020_61135_MOESM1_ESM.pdf]

Supplementary Information for: Generic predictions of output probability based on complexities of inputs and outputs

Kamaludin Dingle[1,2], Guillermo Valle Pérez[2], Ard A. Louis[2]
[1]*Centre for Applied Mathematics and Bioinformatics, Department of Mathematics and Natural Sciences, Gulf University for Science and Technology, Hawally 32093, Kuwait,*
[2]*Rudolf Peierls Centre for Theoretical Physics, University of Oxford, Parks Road, Oxford, OX1 3PU, United Kingdom*

I. ALTERNATIVE WAYS TO DERIVE THE CUMULATIVE BOUND

In the main text an upper bound on the probability $P(x)$ that an output obtains upon uniform random sampling of inputs, is given as

$$P(x) \leq 2^{-a\tilde{K}(x)-b} \tag{1}$$

This bound was introduced in[1].

Here we examine other ways of deriving what are effectively lower bounds on the probability, as expressed in the cumulative bound (8). First consider, as in[1], the function

$$q(x) = \frac{P_0(x)}{P(x)} \tag{2}$$

where $P_0(x) = 2^{-K(x|f,n)+\mathcal{O}(1)}$. Here $q(x)$ measures the ratio of the upper bound of Eq. (1) to the probability $P(x)$ that an output x is generated by random sampling of inputs. Because we work with computable maps, $\sum_x P(x) = 1$, by definition. However, the bound $P_0(x)$ is not normalised, as it is an upper bound on the true probability. One measure of its cumulative tightness is to calculate the expected value of $q(x)$ summed over all inputs, which we call $\mathcal{E}_I$. This can be written as a sum over all outputs, where every output is weighed as $P(x)$:

$$\mathcal{E}_I = \frac{1}{N_I}\sum_{i=1}^{N_I} q(x(p_i)) = \sum_{j=1}^{N_O} P(x_j)q(x_j) = \sum_{j=1}^{N_O} P_0(x_j) \tag{3}$$

By definition of an upper bound, $q(x) \geq 1$ which means that $\mathcal{E}_I = \sum_{x\in O} 2^{-K(x|f,n)+\mathcal{O}(1)} \geq 1$. Interestingly, because $K(x|f,n)$ is a prefix code, $\sum_{x\in O} 2^{-K(x|f,n)} \leq 1$. Therefore $\mathcal{E}_I > 1$ due to the $\mathcal{O}(1)$ terms.

In[1] Markov's inequality was used to derive a lower bound upon uniform random sampling of inputs,

$$\frac{P_0(x)}{\mathcal{E}_I r} \leq P(x) \leq P_0(x) \tag{4}$$

which holds with a probability of at least $1 - \frac{1}{r}$. The upper bound, given approximately by equation (1), always holds of course. We measured $\mathcal{E}_I$ explicitly for the maps in the main text compared to our approximate upper bound and find that typically $\log_{10}\mathcal{E}_I \approx 1$ or 2, which means that the bound is tight on a log scale at least.

Another related way to derive a cumulative bound such as that of Eq (10) from the main text follows a very simple argument. Recall that $\mathcal{D}(f)$ is defined as the set of all outputs x_i that satisfy $(\log_2(P_0(x_i)) - \log_2(P_(x_i))) \geq \Delta$. Recall also that the upper bound is defined as $P_0(x) = 2^{-K(x|f,n)+\mathcal{O}(1)}$. Then we can obtain the bound as follows.

$$\begin{aligned}\sum_{x\in\mathcal{D}(f)} P(x) \leq \sum_{x\in\mathcal{D}(f)} P_0(x)2^{-\Delta} &= \sum_{x\in\mathcal{D}(f)} 2^{-K(x|f,n)+\mathcal{O}(1)-\Delta} \\ &= 2^{-\Delta+\mathcal{O}(1)} \sum_{x\in\mathcal{D}(f)} 2^{-K(x|f,n)} \\ &\leq 2^{-\Delta+\mathcal{O}(1)} \sum_{x} 2^{-K(x|f,n)} \\ &\leq 2^{-\Delta+\mathcal{O}(1)},\end{aligned}$$

where the last line follows from Kraft inequality[2], which applies because $K(x)$ comprise a prefix code. If instead Eq (3) were used for $\sum_x P_0(x) = \mathcal{E}_I$ in the derivation above, then we would obtain

$$\sum_{x\in\mathcal{D}(f)} P(x) \leq \mathcal{E}_I 2^{-\Delta} \tag{5}$$

Although these arguments result in essentially the same bound as the cumulative bound in the main text, the connection with the complexity of inputs is more opaque. However, these derivations highlight other aspects of the bound, such as the role of the $\mathcal{O}(1)$ term in the exponent. Therefore, the two derivations may give insight into the tightness of the looseness of the bound in different situations.

[1] Dingle, K., Camargo, C. Q. & Louis, A. A. Input–output maps are strongly biased towards simple outputs. *Nature communications* **9**, 761 (2018).
[2] Li, M. & Vitanyi, P. *An introduction to Kolmogorov complexity and its applications* (Springer-Verlag New York Inc, 2008).
